# Gastrointestinal, vaginal, nasopharyngeal, and breast milk microbiota profiles and breast milk metabolomic changes in Gambian infants over the first two months of lactation: A prospective cohort study

**DOI:** 10.1097/MD.0000000000031419

**Published:** 2022-11-18

**Authors:** Konstantinos Karampatsas, Amadou Faal, Mustapha Jaiteh, Isabel Garcia-Perez, Sean Aller, Alexander G. Shaw, Aleksandra Kopytek, Adam A. Witney, Kirsty Le Doare

**Affiliations:** a Centre for Neonatal and Paediatric Infection, St George’s, University of London, London, UK; b Medical Research Council The Gambia at London School of Hygiene and Tropical Medicine, Fajara, The Gambia; c Division of Digestive Diseases, Department of Metabolism, Digestion and Reproduction, Faculty of Medicine, Imperial College London, London, UK; d Department of Infectious Disease Epidemiology, Imperial College London, London, UK; e Medical Research Council Uganda, Virus Research Institute, Uganda.

**Keywords:** breast feeding, gastrointestinal microbiome, metabolome, respiratory microbiome

## Abstract

Microbiota composition in breast milk affects intestinal and respiratory microbiota colonization and the mucosal immune system’s development in infants. The metabolomic content of breast milk is thought to interact with the microbiota and may influence developing infant immunity. One hundred seven Gambian mothers and their healthy, vaginally delivered, exclusively breastfed infants were included in our study. We analyzed 32 breast milk samples, 51 maternal rectovaginal swabs and 30 infants’ rectal swabs at birth. We also analyzed 9 breast milk samples and 18 infants’ nasopharyngeal swabs 60 days post-delivery. We used 16S rRNA gene sequencing to determine the microbiota composition. Metabolomic profiling analysis was performed on colostrum and mature breast milk samples using a multiplatform approach combining 1-H Nuclear Magnetic Resonance Spectroscopy and Gas Chromatography-Mass Spectrometry. Bacterial communities were distinct in composition and diversity across different sample types. Breast milk composition changed over the first 60 days of lactation. α-1,4- and α-1,3-fucosylated human milk oligosaccharides, and other 33 key metabolites in breast milk (monosaccharides, sugar alcohols and fatty acids) increased between birth and day 60 of life. This study’s results indicate that infant gut and respiratory microbiota are unique bacterial communities, distinct from maternal gut and breast milk, respectively. Breast milk microbiota composition and metabolomic profile change throughout lactation. These changes may contribute to the infant’s immunological, metabolic, and neurological development and could consist the basis for future interventions to correct disrupted early life microbial colonization.

## 1. Introduction

It has been proposed that human gut microbiota play a significant role in maintaining lifelong health.^[1]^ Perturbations in the infant gut microbiota composition have been associated with increased susceptibility to various diseases early in life.^[1,2]^ Less is known about the respiratory microbiota, but studies have identified disruptions in the respiratory microbiota in the first months of life to predict future respiratory health.^[3]^

The initial colonization and establishment of microbiota in infancy constitute a complex and dynamic process, influenced by multiple factors, such as mode of delivery,^[4]^ gestational age at birth,^[5]^ type of feeding,^[6]^ antibiotic treatment,^[7]^ maternal diet,^[8]^ environmental exposures,^[9]^ and host genetics.^[10]^

Human milk is considered a significant contributory factor to the development of the infant gut microbiota either by directly seeding the infant gut,^[11,12]^ or through the role of human milk oligosaccharides (HMOs) in promoting the growth of *Bifidobacterium* species in the human gut.^[13]^ The same may be true of the respiratory microbiome with breastfeeding potentially affecting patterns of colonization.^[14,15]^ Also, it has been recently recognized that the breast milk metabolomic profile changes over time in the transition from colostrum to mature milk to promote the growing infant’s immunological maturation and neurological development.^[16]^ However, the interplay between the breast milk metabolome and the infant microbiome is, as yet, unknown.

In this study, we used 16S rRNA gene sequencing to characterize the gut and nasopharyngeal microbiome in healthy, vaginally delivered infants from the Gambia, together with the breast milk and rectovaginal microbiome of their mothers. In addition, we assessed the metabolomic changes of breast milk over the first two months of life in the same cohort.

## 2. Materials and methods

### 2.1. Study population

Our Gambian samples were nested within a large longitudinal cohort study to examine risk factors for group B streptococcus colonization in Gambian mothers and their infants.^[17]^ The study was conducted between January 15, 2014, and January 31, 2015, in Faji Kunda Hospital and Jammeh Foundation for Peace Hospital, two public health centers in Gambia’s urban coastal Banjul region with 12,500 births each year. These two centers were chosen because they represent the antenatal services that Gambian women typically have. The inclusion criteria were aged 18 to 45 years, negative HIV test during pregnancy, written informed consent obtained from the infant’s mother, ability to comply with the study procedures as judged by a member of the research team, low-risk pregnancy defined as no evidence of pre-eclampsia, cardiomyopathy, maternal gestational diabetes, placental previa, twin pregnancy or any other condition or situation that substantially increased the risk of pregnancy based on the investigator’s clinical judgement, meaning that participation would not be in the best interests of the infants. Women were not recruited if they were planning to move outside the study area for at least the first 12 weeks following delivery (preventing follow-up visits), were enrolled in other studies requiring blood/breast milk sampling or swabs, were not planning to breastfeed, developed complications of delivery (pre-eclampsia, antepartum hemorrhage, cesarean section), or if the infant was born below 32 weeks’ gestation as assessed by Ballard score, had a birth weight less than 2.5 kg, diagnosed with congenital abnormalities requiring prolonged hospital stay (>48 hours), showed symptoms or signs of significant illness or infection at birth, required resuscitation and intensive care, or for any other reason that would prevent the study endpoints being assessed in the infant effectively as judged by the investigator. From the 750 mother-infant pairs recruited into the main study, 107 mothers and their infants were randomly included in the microbiota study.

### 2.2. Sample collection

We collected breast milk samples, maternal rectovaginal swabs and infants’ rectal swabs at birth (D0). We also collected breast milk samples and infants’ nasopharyngeal swabs 60 days post-delivery (D60). Swabs (Copan, UK) were collected in skim-milk tryptone, glucose glycerol (STGG) medium, refrigerated at 4°C and transported to the laboratory within 6 hours, to be vortexed and stored at –70°C. For the breast milk collection, mothers were requested to wash their hands with soap and wipe their breasts with sterile cotton wool and 0.02% chlorhexidine before hand expressing a milk sample from each breast into separate sterile containers. After collection, milk samples were refrigerated at 4°C and transported to the laboratory within 4 hours. Then the samples were spun at 3000 g for 30 minutes to remove lipids and were frozen at –70°C.

### 2.3. Microbiota analysis

#### 2.3.1. DNA extraction, library preparation, and sequencing.

Samples were transferred to Imperial College London for analysis. DNA was purified using Fast DNA™ SPIN Kit for Soil (MP BIOMEDICALS, Santa Ana, CA) according to the manufacturer’s instructions.^[18]^ The microbiota composition was established by conducting a nested polymerase chain reaction to amplify first V3–V5^[19]^ and then V4 hypervariable regions of the 16S rRNA gene.^[20]^

#### 2.3.2. Sequence processing and microbial species abundance estimates.

Amplicon sequence data were analyzed using Mothur is Open-source, platform-independent, community-supported software v1.43.0^[21]^ according to the Mothur MiSeq SOP.^[22]^ Overlapping sequencing reads were merged into contigs, cleaned and aligned to a V4 restricted version of the SILVA reference database (version 138).^[23]^ Sequences were clustered into operational taxonomic units at 97% similarity using the OptiClust algorithm and classified using both the SILVA and GreenGenes (version 13_8_99) reference databases.^[24]^ Any contaminating taxa identified at significant levels within the negative controls were filtered out of the sample set; these included members of the *Rhizobiales* order and *Sericytochromatia*. Samples were “normalized” by sub-sampling at 3000 sequences to balance sample inclusion with sufficient coverage (mean Good coverage = 98.1%, standard deviation 0.008%).

### 2.4. Metabolomics

Metabolic profiling analyses of breast milk samples were conducted using established 1-H-Nuclear Magnetic Resonance (1H-NMR) and Gas chromatography-mass spectrometry (GC-MS) metabolic profiling analysis methods. These methods have been described in detail in previous publications.^[25–27]^

### 2.5. Statistical analyses

Differences in alpha diversity metrics were compared using the Shannon’s diversity index measure of community richness, observed species and the Chao1 index, and differences in beta diversity using the Bray-Curtis distance measure of community dissimilarity. Alpha diversity was explored using the Shapiro test for normality, with significant differences calculated by the non-normal distribution Wilcox test. *P*-values were adjusted for multiple comparisons using the Benjamini-Hochberg method. When comparing the relative abundance of genera between sample groups, we applied an arbitrary threshold of > 10% relative abundance in at least one sample for each group to generate a hypothesis. Multivariable statistical analysis was performed on the 1H-NMR and GC-MS acquired data. Repeated Measures Monte Carlo Cross-Validated Partial Least Squares (RM-MCCV-PLS) analysis was performed on each of the data sets. Linear Regression analysis of 1H-NMR and GC-MS breast milk profiles was performed against diversity indices (observed species, Bergerparker, Shannon, Chao, Simpson) of the most abundant genera detected in maternal and infant swabs and breast milk, adjusted using the Storey-Tibshirani False Discovery Rate (FDR) and corrected for confounding factors (sex, ethnic group). Metabolites with adjusted pFDR values  <0.01 were considered significant and were subsequently visualized in a Manhattan plot. All statistical analyses were completed in R (version 3.6.1).

### 2.6. Ethical approval

The study was reviewed and approved by the Gambian Government/Medical Research Council Joint Ethics Committee (application SCC 1350.v4). All research was performed following the relevant guidelines and regulations, in accordance with the Declaration of Helsinki Ethical Principles and Good Clinical Practices.

### 2.7. Data availability

All data and metadata are openly available at St George’s University figshare data deposit (https://doi.org/10.24376/rd.sgul.14045945). Sequence data have been submitted to the European Nucleotide Archive database (https://www.ebi.ac.uk/ena/browser/home) with accession number PRJEB41404.

## 3. Results

### 3.1. Study population

We successfully performed 16S rRNA gene sequencing on breast milk samples and swabs from 142 participants (35 mother–infant pairs, 72 mothers only). Demographic and clinical characteristics are reported in Table 1. Pregnancy was uneventful for most women. Seven (6.5%) women had a urinary tract infection, 2 (1.9%) had pneumonia and 1 (0.9%) had malaria. In addition, 17 (15.9%) participants received antibiotics, of which 16 (94%) had a course of amoxicillin with a mean interval between initiation of antibiotics and birth of 21 (range 0–157) days. Thirty six (33.6%) women were colonized with Group B Streptococcus at the time of delivery. Intrapartum antibiotic prophylaxis was not routinely given in the Faji Kunda area during the study unless the woman had fever in labor. All infants were born by vaginal delivery and were exclusively breastfed for the full duration of the study. During the first two months of life, 6 (17.1%) infants attended a medical facility at a median age of 15 (IQR 9) days. Of these 6 infants, 2 had an unspecified skin rash, 1 infected umbilicus, 1 fever of unknown cause and 1 jaundice. Four of them received amoxicillin or cloxacillin.

**Table 1:** Demographic and Clinical Characteristics of Gambian mothers and infants from whom milk samples and swabs were successfully collected and sequenced.

**Women [N = 107]**	
Age in years: median (IQR)	24 (20–29)
Gravida: median (IQR)	2 (1–4)
Parity: median (IQR)	1 (0–3)
Weight in kg: median (IQR)	64.6 (58–75.5)
Delivery mode: % vaginal	100
Antibiotics during pregnancy: % received antibiotics	15.8
Hemoglobin in g/dL: median (IQR)	11 (10.1–11.4)
Group B Streptococcal colonization at delivery: % positive	33.7
**Infants** [N = 35]	
Gestation in weeks: median (IQR)	38 (36–40)
Birth weight in kg: median (IQR)	3.4 (3.0–3.6)
Sex % female	52.3

### 3.2. Composition and diversity of the microbiota in infants and mothers

After sub-sampling at a depth of 3000 sequences (see Figure, Supplemental Digital Content 1, http://links.lww.com/MD/H797, which illustrates the sequencing reads per sample type), the final microbiome analysis included 51 maternal rectovaginal swabs, 30 infant rectal swabs and 32 breast milk samples (colostrum) collected on D0; and 18 infant nasopharyngeal swabs and 9 breast milk samples (mature breast milk) collected on D60 (see Figure, Supplemental Digital Content 2, http://links.lww.com/MD/H798, which is the flowchart of samples collected from study participants).

### 3.3. Overall taxonomic and alpha diversity analyses

**Figure 1.**
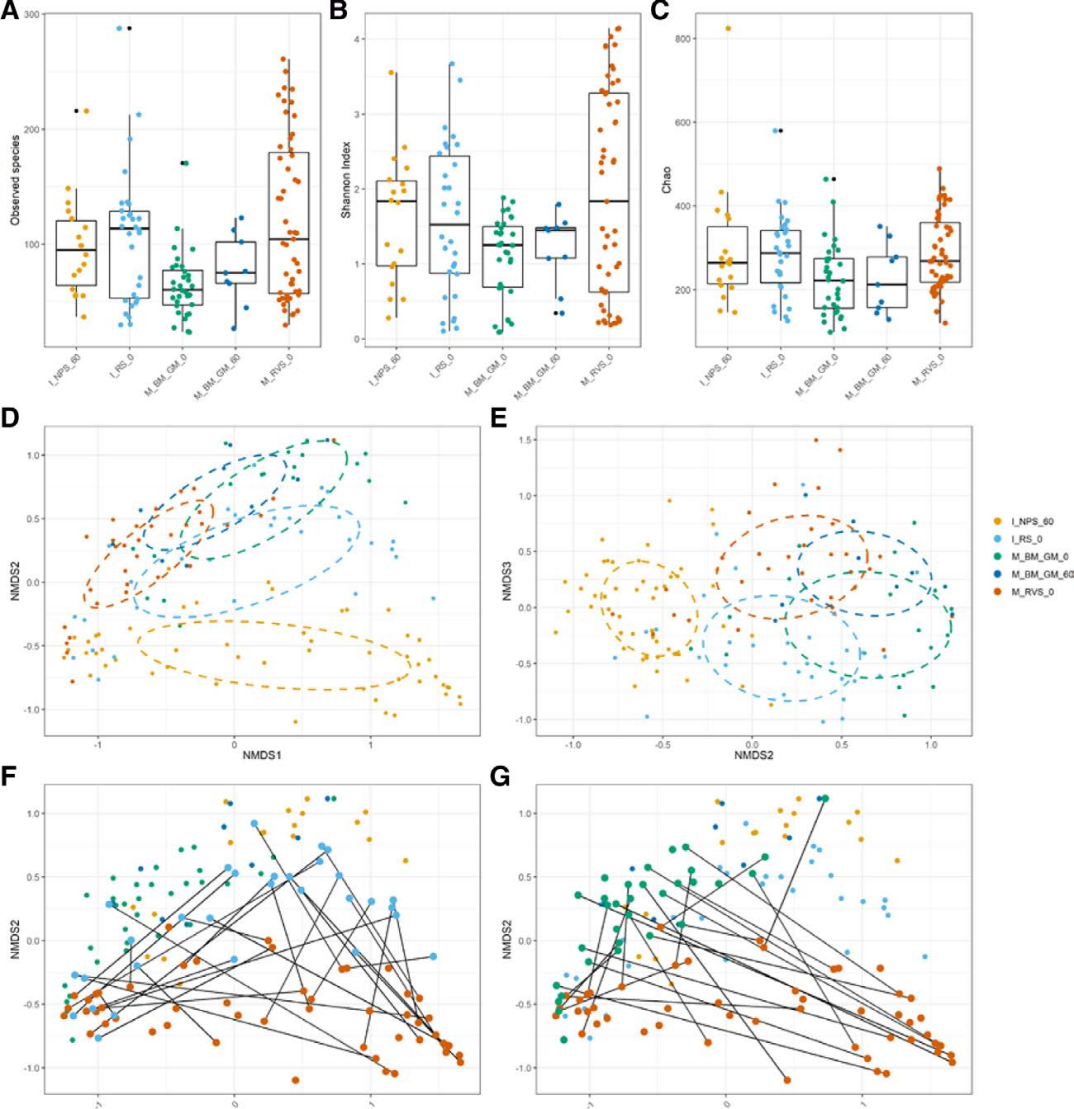
Alpha diversity of maternal and infant microbiomes across different body sites, determined using (A) observed species, (B) Shannon index, or (C) Chao1 index, (D) beta diversity of maternal and infant microbiomes across different body sites, based on NMDS analysis (stress = 0.11): NMDS1 v NMDS2, (E) NMDS2 v NMDS3, (F) maternal rectovaginal swabs versus infant rectal swabs at birth from mother/infant pairs, and (G) maternal rectovaginal swabs versus maternal colostrum at birth from the same participants. Statistics: Shapiro–Wilk normality test was performed. Kruskal-Wallis rank-sum test and pairwise Wilcoxon rank-sum test were performed to assess significance between groups. Multiple testing corrections were performed using the Benjamini-Hochburg procedure. I_NPS_60: Infant nasopharyngeal swabs day 60; I_RS_0: Infant rectal swabs at birth; M_BM_GM_0: Breast milk at birth (colostrum); M_BM_GM_60: Breast milk on D60 (mature breast milk); M_RVS_0: Maternal rectovaginal swabs at birth. NMDS = non-metric multidimensional scaling.

We found that colostrum samples harbored significantly fewer observed species compared to maternal rectovaginal swabs (*P* = .004) and infant rectal swabs at birth (*P* = .008) (Fig. 1A). Shannon diversity and Chao1 index of microbiota did not differ across different body sites (Fig. 1B and C).

### 3.4. Beta diversity and taxa relative abundance analyses

In a Non-metric Multidimensional Scaling analysis (NMDS) of Bray-Curtis dissimilarity, microbiota clustered for both body site and collection time point (Fig. 1D and E). At birth, microbiota in infant rectal swabs was distinct from both colostrum and maternal rectovaginal swabs. On D60, nasopharyngeal and mature breast milk microbial communities were also separated into 2 distinct groups. In addition, colostrum and mature breast milk samples had distinct microbiota compositions. Similarly, when only samples collected from the same mother-infant pairs were compared, maternal rectovaginal microbiota clustered separately from infant gastrointestinal (Fig. 1F) and breast milk microbiota (Fig. 1G).

**Figure 2.**
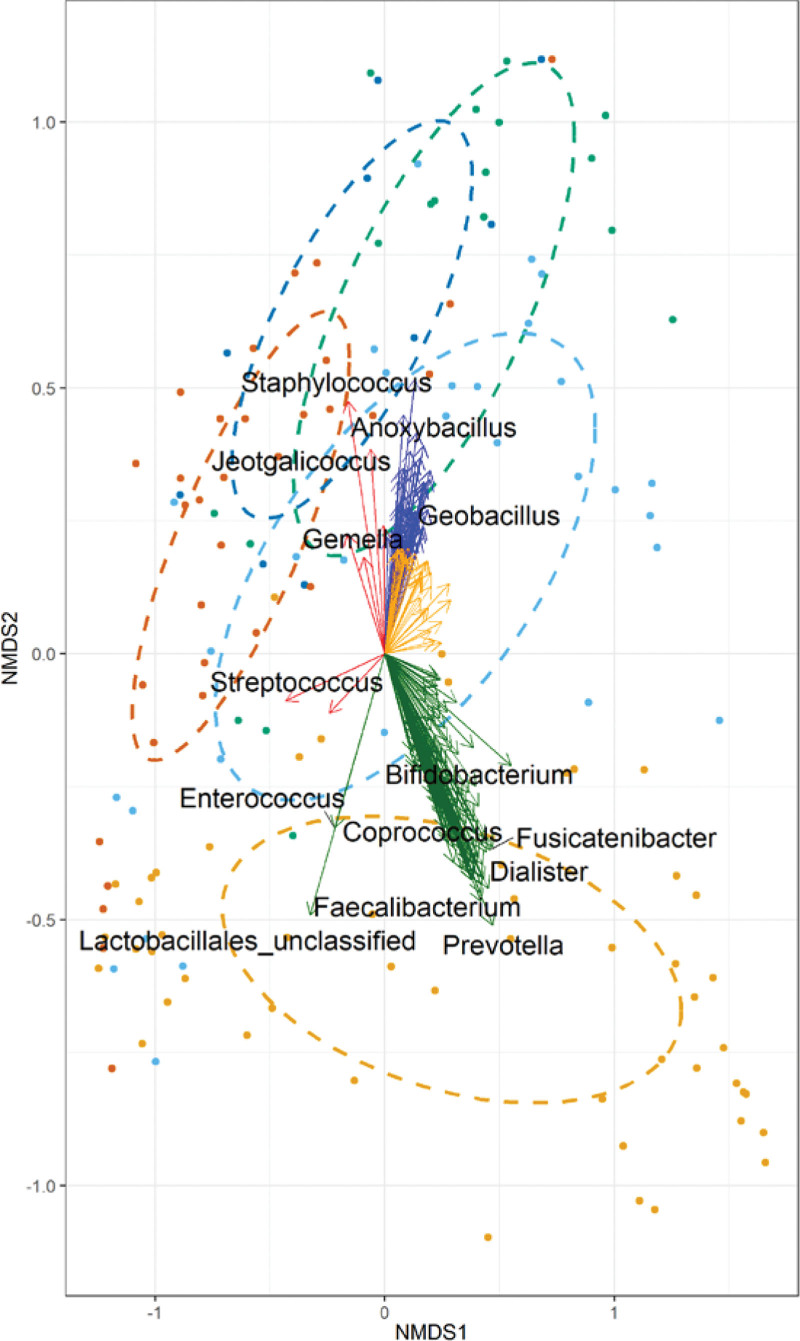
Beta diversity of maternal and infant microbiomes across different body sites, based on non-metric multidimensional scaling (NMDS) analysis (using the R vegan metaMDS function) with significant species overlaid (using the vegan envfit function, *P* < .05). The arrows represent species with significant correlation with the community structure and are coloured by direction. Previously described notable taxa are labeled. I_NPS_60: Infant nasopharyngeal swabs day 60; I_RS_0: Infant rectal swabs at birth; M_BM_GM_0: Breast milk at birth (colostrum); M_BM_GM_60: Breast milk on D60 (mature breast milk); M_RVS_0: Maternal rectovaginal swabs at birth.

Two hundred eighty bacterial taxa correlated with the community structure (*P* < .05) in the different sample types. We have listed some notable ones that were described in previous studies (Fig. 2). Microbiota in maternal rectovaginal swabs were split into 2 sub-groups; the first was associated with fecal microbes like *Prevotella*, *Faecalibacterium*, *Dialister*, *Coprococcus* and *Fusicatenibacter* and the second one with predominantly vaginal ones like *Lactobacillales*. Species belonging to the *Bifidobacterium* genus were associated with the infant gut microbiota. *Streptococcus* species were associated with D0 breast milk samples, whereas *Staphylococcus* and *Gemella* with D60 breast milk samples. Bacterial genera like *Anoxybacillus*, *Jeotgalicoccus* and *Geobacillus* were associated with nasopharyngeal microbiota (Fig. 2).

**Figure 3.**
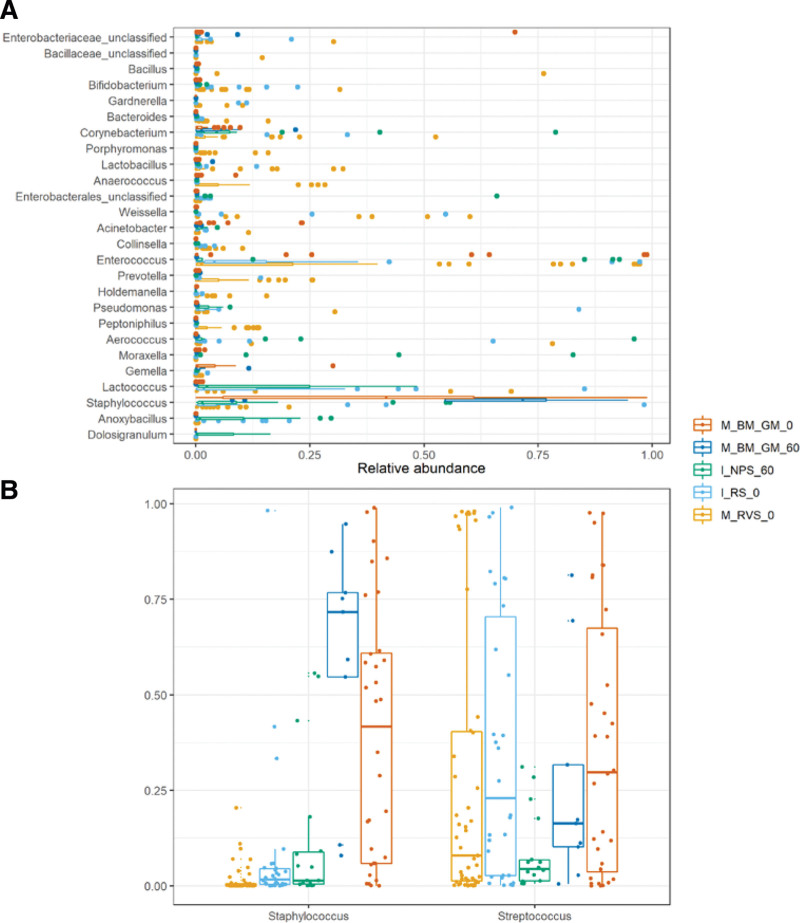
(A) Relative abundance of genera, with greater than 10% relative abundance in at least one sample for each group. (B) Relative abundance of Staphylococcus and Streptococcus for each Group. I_NPS_60: Infant nasopharyngeal swabs day 60; I_RS_0: Infant rectal swabs at birth; M_BM_GM_0: Breast milk at birth (colostrum); M_BM_GM_60: Breast milk on day 60 (mature breast milk); M_RVS_0: Maternal rectovaginal swabs at birth.

Twenty-four genera were found to significantly differ between sample groups when we applied a cut off of > 10% relative abundance in at least 1 sample for each group (Fig. 3A). When comparing the relative abundance of the 2 most relevant genera (*Streptococcus* and *Staphylococcus*) that showed a significant correlation in beta diversity analysis (Fig. 3B), we found higher levels of *Staphylococcus* in breast milk’s microbiota compared to the nasopharyngeal microbiota (*P* < .001).

### 3.5. Metabolic phenotyping of breast milk HMOs

All the breast milk samples collected on D0 (n = 70) and D60 (n = 68) were included in the metabolomic analysis.

### 3.6. 1H-NMR

**Figure 4.**
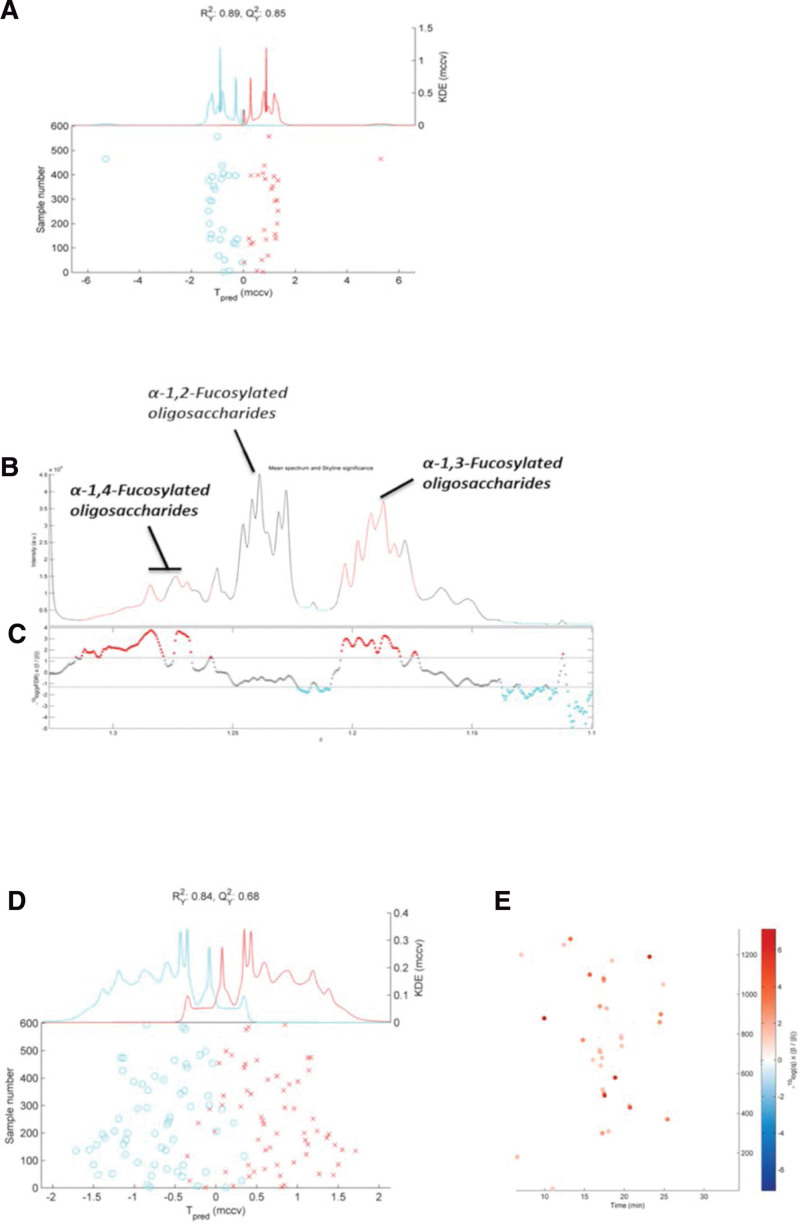
1H-NMR RM-MCCV-PLS scores plot model comparing Breast milk samples at D0 (Ciano dots) with D60 of life (red crosses). The top part of the panel gives the Kernel Density Estimate (KDE) of each group’s predicted scores. (A) The bottom part shows the predicted scores (Tpred) from MCCV for each sample. (B) Fragment of the average 600 MHz 1H-NMR breast milk spectrum to visualized some identified labeled metabolites. (C) Manhattan plot showing -log10(q) × sign of regression coefficient (*β*) of the RM MCCV–PLS model for the 16,000 spectral variables. Red peaks represent the variables that significantly increased over time (D60), and blue peaks represent the variables that significantly decreased over time (D0). (D) GC-MS RM-MCCV-PLS scores plot model comparing Breast milk samples at D0 (Ciano dots) with D60 of life (red crosses). The top part of the panel gives the KDE of each group’s predicted scores. The bottom part shows the predicted scores (Tpred) from MCCV for each sample. (E) Manhattan plot showing -log10(q) × sign of regression coefficient (*β*) of the RM MCCV–PLS model for the 16,000 spectral variables. Red dots represent the variables (metabolites) that significantly increased over time (D60, and blue dots represent the variables that significantly decreased over time (D0). A *P*-value was calculated for each variable. *P*-values were adjusted for multiple testing using the Storey-Tibshirani False Discovery Rate (FDR, q-value). KDE = Kernel Density Estimate, RM MCCV–PLS = repeated measures Monte Carlo cross-validated partial least squares analysis.

Data from the 1H-NMR spectroscopic analysis were used to construct an RM-MCCV-PLS model (Fig. 4A). Breast milk samples clustered according to the time of collection (D0 vs D60). We found that *α*-1,4-fucosylated oligosaccharides and *α*-1,3-fucosylated oligosaccharides significantly increased over time (Fig 4B and C), whereas *α*-1,2-fucosylated oligosaccharides remained the same over time.

### 3.7. GC-MS

Repeated measures Monte Carlo cross-validated partial least squares analysis performed on the GC-MS data also revealed separate clustering of colostrum and mature breast milk samples (Fig. 4D). This was mainly due to an increase in the relative abundance of 33 metabolites, such as monosaccharides, sugar alcohols, fatty acids, and fatty acid esters (Fig. 4E, Table 2).

**Table 2:** Increase in the relative abundance of 33 breast milk metabolites between birth day and day 60 of life.

Metabolite	Metabolite class	*P* value
Palmitic acid	Fatty acids	<.001
Lauric acid	Fatty acids	<.001
Myristic acid	Fatty acids	.02
Capric acid	Fatty acids	.03
Trans-13-octadecenoic acid	Fatty acids	.05
Dioctyl phthalate	Phthalic acid and derivatives	<.001
Lactobionic acid	Sugar acids	<.001
Glucoheptonic acid	Sugar acids	.02
Lactobionic acid	Sugar acids	.06
m-toluic acid	Organic acids	.05
Glycolic acid	Organic acids	.05
Methyl Stearate	Fatty acid esters	.04
D-allose	Monosaccharides	<.001
Rhamnose	Monosaccharides	<.001
Talose	Monosaccharides	<.001
d (+)altrose	Monosaccharides	.001
d-mannose	Monosaccharides	.002
d-allose	Monosaccharides	.02
l-sorbose	Monosaccharides	.03
Psicose	Monosaccharides	.05
Beta-gentiobiose	Disaccharides	<.001
Cellobiose	Disaccharides	<.001
Methyl beta-D-galactopyranoside	Saccharides	.001
Allo-inositol	Carbocyclic sugars	.002
Sedoheptulose anhydride monohydrate	Anhydro sugars	.01
Glycerol	Sugar alcohols	<.001
1,5-Anhydro-D-sorbitol	Fatty alcohols	.009
conduritol epoxide	Fatty alcohols	.02
2,3-Butanediol	Alcohols	.01
Xanthotoxin	Coumarins and derivatives	<.001
Ribulose-5-phosphate	Sugar phosphates	.05
Glycerol 1-phosphate	Sugar phosphates	.05
Eicosane	Alkanes	.06

Finally, linear regression analysis showed no significant correlations between the 1H-NMR breast milk spectra and the GC-MS breast milk profiles and bacterial diversity indexes such as observed species, Bergerparker, Shannon, Chao, Simpson from the maternal and infant swabs. Similarly, no significant correlations were found in a linear regression analysis between breast milk metabolomic profiles and the most abundant genera differentially detected in the maternal rectovaginal swabs and infant rectal swabs.

## 4. Discussion

To our knowledge, this is the first study to report distinct bacterial communities in both composition and diversity from breast milk (D0 and D60), maternal rectovaginal swabs (D0), infant rectal swabs (D0) and infant nasopharyngeal swabs (D60) in vaginally-delivered infants from the Gambia. This is also the first study that assessed breast milk’s metabolomic changes during the first 2 months of lactation in the Gambia. We found changes in the relative abundance of HMOs and other key metabolites that might contribute to the healthy infant’s immunological, metabolic, and neurological development. However, we found no significant correlations between breast milk metabolomic profiles and the maternal rectovaginal and infant gut microbiome at birth.

In our study, infants’ gut microbiota differed from maternal rectovaginal microbiota at birth. This finding is in accordance with differences between maternal and infant microbiota within 24 hours of delivery reported in a recent study of mother-to-infant microbial transmission.^[11]^ This is not to dispute the paramount importance of vertical transmission through the birth canal or maternal skin depending on the mode of delivery, but to highlight that the results of this seeding event are only manifested a few days or weeks after birth.^[28,29]^

We found significant changes in the composition and diversity of microbial communities in breast milk through lactation, in agreement with previous studies.^[30,31]^ *Staphylococcus* was a common component of breast milk microbiota that increased with lactation. Typical oral cavity genera such as *Streptococcus* and *Gemella* were also prevalent in breast milk samples, as described before.^[32,33]^ This is in keeping with previous reports suggesting that a “core” breast milk microbiota consist of *Streptococcus* and *Staphylococcus*,^[34,35]^ despite inter-individual variability and observed differences across populations.^[31,36]^

In addition, we found that nasopharyngeal and breast milk microbiota clustered separately on D60. The infant nasal microbiota has been previously shown to resemble the skin microbiota composition in the early weeks of life, probably due to transmission from the mother’s skin during breastfeeding and gradually shifting towards a respiratory microbiota by the age of 3 months.^[3,15]^ In our cohort, genera of the phylum Firmicutes, such as *Anoxybacillus*, *Jeotgalicoccus*, and *Geobacillus*, although in low abundance, were over-represented. The data on the role of *Anoxybacillus* are contradictory. An increased presence of *Anoxybacillus* in the nasopharynx has recently been shown to raise the risk of respiratory tract infections in Venezuelan infants < 2 years of age.^[37]^ In contrast, a previous study suggested that decreased abundance of *Anoxybacillus* was related to overgrowth of bacterial pathogens causing otitis media [29].^[38]^ The presence of these soil bacteria that thrive in humid environments in the respiratory microbiota of 2-month-old infants probably reflects their living environment. Except for 1 infant, the rest (20/21, 95%) were born during the green season (mid-June–end of October) with average humidity of 76-86%. This might have contributed to our findings.

We found changes in the relative abundance of fucosylated HMOs between colostrum and mature breast milk. Overall, *α*-1,3- and α-1,4- fucosylated oligosaccharides significantly increased throughout lactation, whereas α-1,2- fucosylated oligosaccharides remained stable. A recent systematic review has shown that the concentration of *α*-1,3- fucosylated oligosaccharides significantly increased throughout lactation^[39]^ and then gradually decreased after weaning.^[40]^ This variation across lactation stages suggests that environmental factors might play a role in the activity of fucosyltransferases encoded by the Lewis and Secretor gene alleles.^[41]^

Changes in the breast milk composition over time reflect the changing needs of the growing infant. In support of this, we found a rise in the relative concentrations of monosaccharides, glycerol and short and medium-chain fatty acids in the first 60 days of lactation, in accordance with previous metabolomic studies of milk maturation.^[25,26,42–46]^ Although the biological importance of these changes is not yet fully understood, most of these metabolites play a role in the immunological maturation and brain development of the growing infant.^[47]^

Non-microbial components of breast milk might shape the breast milk and infant respiratory and gut microbiota composition.^[48]^ Despite the biological plausibility of this hypothesis, we did not confirm it in our study. A recent longitudinal birth cohort study also found no direct correlation between breast milk microbiota and other milk elements, such as HMOs and fatty acids.^[49]^ Further studies are required to address these complex interactions.

The clinical relevance of breast milk HMOs and the other non-microbial components in preventing infectious diseases in neonates and young infants is increasingly recognized. HMOs promote the development of *Bifidobacterium* species that inhibit the growth of pathogens.^[50,51]^ Supplementing human milk with generic or specific HMOs to reduce the risk of necrotizing enterocolitis in premature infants showed promising results in rat models^[52]^ that were replicated in a study of human infants.^[53]^ Like the gut microbiota,^[54]^ modulation of respiratory microbiota by promoting colonization of the nasopharynx by beneficial bacteria can be achieved by the administration of probiotics.^[55,56]^ There is also an increasing interest in the role of prebiotics in the prevention and treatment of respiratory disease. Administration of short-chain fatty acids in pregnant mice suppressed allergic airways disease in their offspring.^[57]^ Oral administration of oligosaccharide-based prebiotics in preterm infants reduced the number of viral respiratory tract infections.^[58]^

There are limitations to our study. In many cases, the quality of the swabs was sub-optimal for analysis, resulting from the challenges of conducting microbiome research in such a setting. We set a sub-sampling depth of 3000 sequences per sample to balance sample inclusion with sufficient coverage, but reducing the number of samples in the final analysis. This is always a major challenge when using low-biomass samples like breast milk. Surprisingly, we found very low abundances of *Bifidobacterium* in the infant rectal swabs collected in our study. There are two likely causes for this finding. First, we sequenced our samples using the V4 region, a method shown to underestimate the presence of *Bifidobacterium* in metagenomic samples due to higher guanine-cytosine content in this region.^[59]^ Second, we used rectal swabs that are more likely to pick up taxa that are aerotolerant and can reside in the skin, instead of stool samples, which may allow sampling of the gut where obligate anaerobes are more likely to reside. Collection of nasopharyngeal samples only during the green season adds further caution regarding the generalizability of our findings of nasal infant microbiota.^[60]^ The interpretation of metabolomic profiling is also limited by the lack of information on the participants’ dietary intake. However, the diet in the Gambia is fairly homogeneous, consisting of rice, maize and bean staples with limited fish or meat. Furthermore, in our study, 10 rectovaginal swabs from women who received antibiotics during pregnancy were included in the final analysis. Although the association between a lower intestinal microbiome diversity and intrapartum antibiotics is well recognized,^[61]^ maternal antibiotic treatment during pregnancy might be causing only short-term perturbations of the composition of infant intestinal microbiome that does not last beyond the age of 1 month.^[62]^ Given that the mean interval between cessation of antibiotics and delivery was 51 days (range 7–108) for these women, the overall effect of antibiotics on the microbiome composition is uncertain. Finally, we included healthy, breastfed infants in the study. However, we did not investigate for congenital infections other than HIV, immune deficiencies and metabolic disorders, since most of these tests were not available in the study area. No infants died during the follow-up visits, and we did not identify any culture-confirmed sepsis. However, some infants might have had other less invasive infections or medical conditions for which they did not seek medical assistance.

In conclusion, we quantified the microbial composition and compared it across subjects and body sites in healthy mother-infant pairs in the Gambia. We confirmed that breast milk microbiota composition and metabolomic profile change throughout lactation. A greater understanding of the interplay between various molecules, cells, and non-microbial components in breast milk will support future interventions to correct disrupted early life gut and nasopharyngeal colonization.

## Author contributions

**Conceptualization:** Isabel Garcia-Perez, Alexander G. Shaw, Kirsty Le Doare.

**Data curation:** Konstantinos Karampatsas, Adam A. Witney, Kirsty Le Doare.

**Formal analysis:** Konstantinos Karampatsas, Isabel Garcia-Perez, Sean Aller, Alexander G. Shaw, Aleksandra Kopytek, Adam A. Witney.

**Funding acquisition:** Kirsty Le Doare.

**Investigation:** Amadou Faal, Mustapha Jaiteh.

**Methodology:** Isabel Garcia-Perez, Alexander G Shaw, Adam A Witney, Kirsty Le Doare.

**Software:** Adam A Witney.

**Supervision:** Adam A Witney, Kirsty Le Doare.

**Writing – original draft:** Konstantinos Karampatsas.

**Writing – review & editing:** Konstantinos Karampatsas, Amadou Faal, Mustapha Jaiteh, Isabel Garcia-Perez, Sean Aller, Alexander G. Shaw, Aleksandra Kopytek, Adam A. Witney, Kirsty Le Doare.

## Supplementary Material
