# Supplementary figures and images for: Gastrointestinal, vaginal, nasopharyngeal, and breast milk microbiota profiles and breast milk metabolomic changes in Gambian infants over the first two months of lactation: A prospective cohort study
Source: Medicine (Baltimore). 2022 Nov 18;101(46):e31419. doi: 10.1097/MD.0000000000031419 (PMC9678627; doi:10.1097/MD.0000000000031419)

## Slide 1
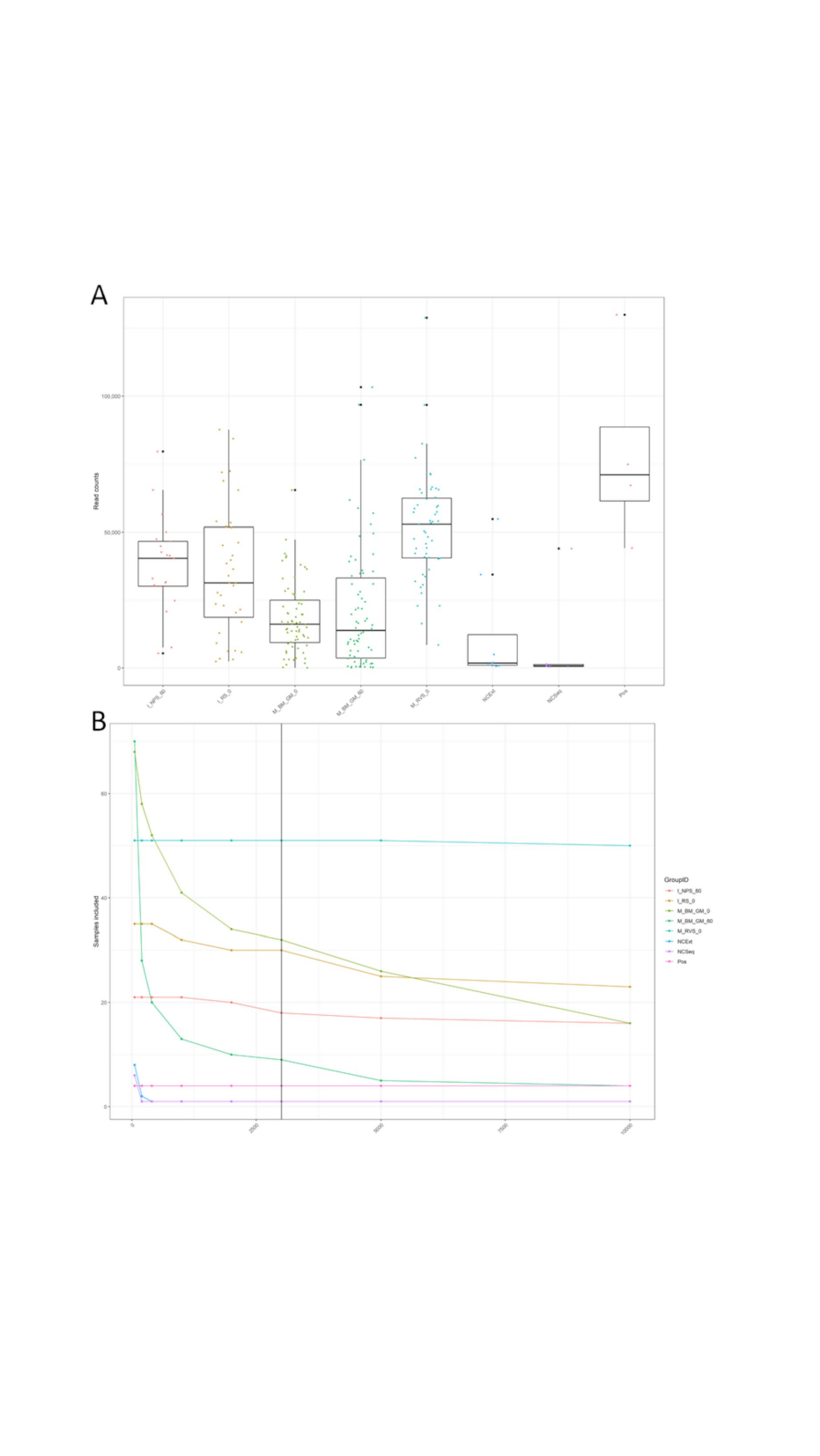

Supplement: Supplementary file 1 [file medi-101-e31419-s001.ppt]

## Slide 1
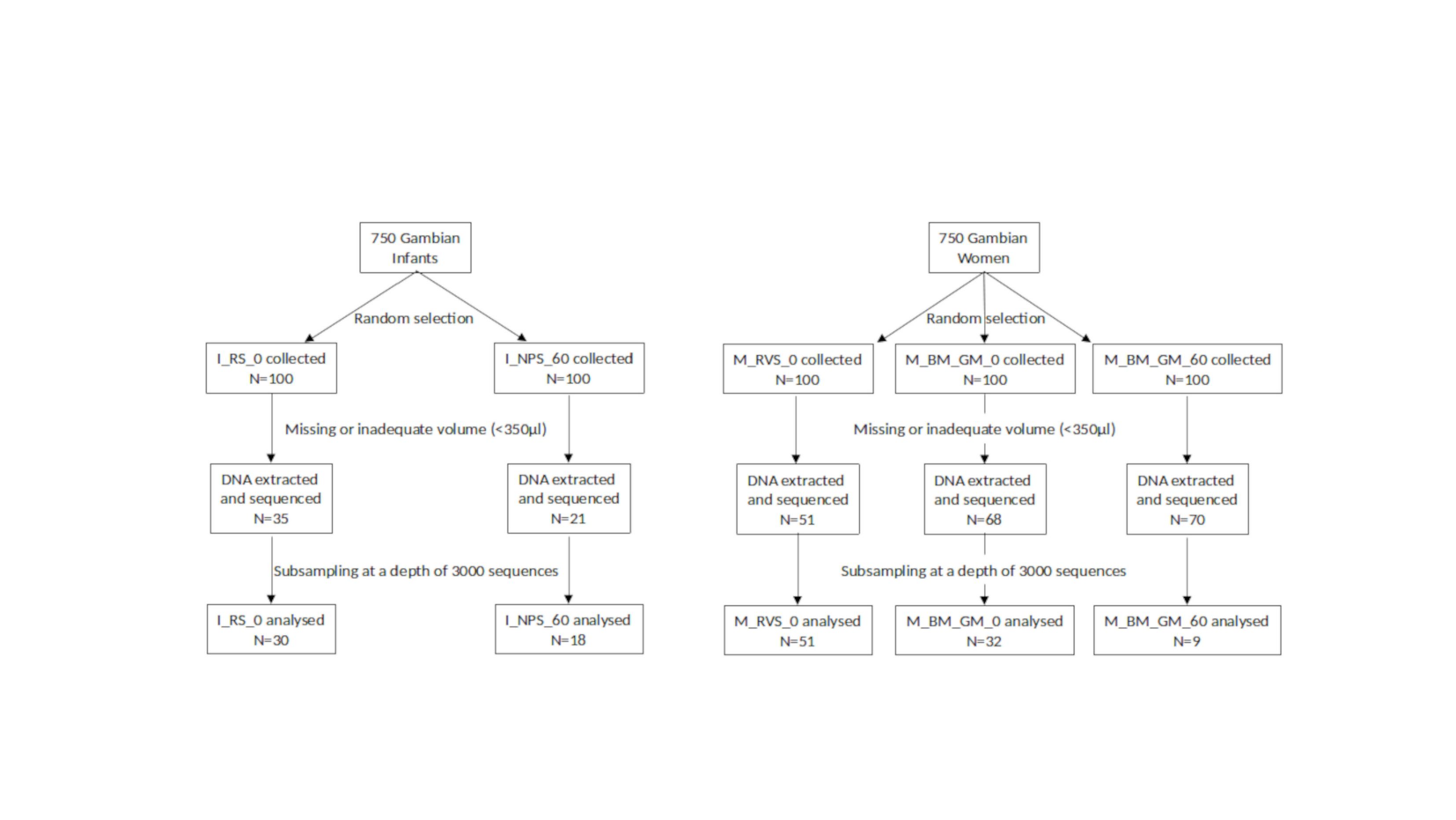

Supplement: Supplementary file 2 [file medi-101-e31419-s002.ppt]
